# Supplementary figures and images for: Loading history changes the morphology and compressive force-induced expression of receptor activator of nuclear factor kappa B ligand/osteoprotegerin in MLO-Y4 osteocytes
Source: PeerJ. 2020 Nov 9;8:e10244. doi: 10.7717/peerj.10244 (PMC7659647; doi:10.7717/peerj.10244)

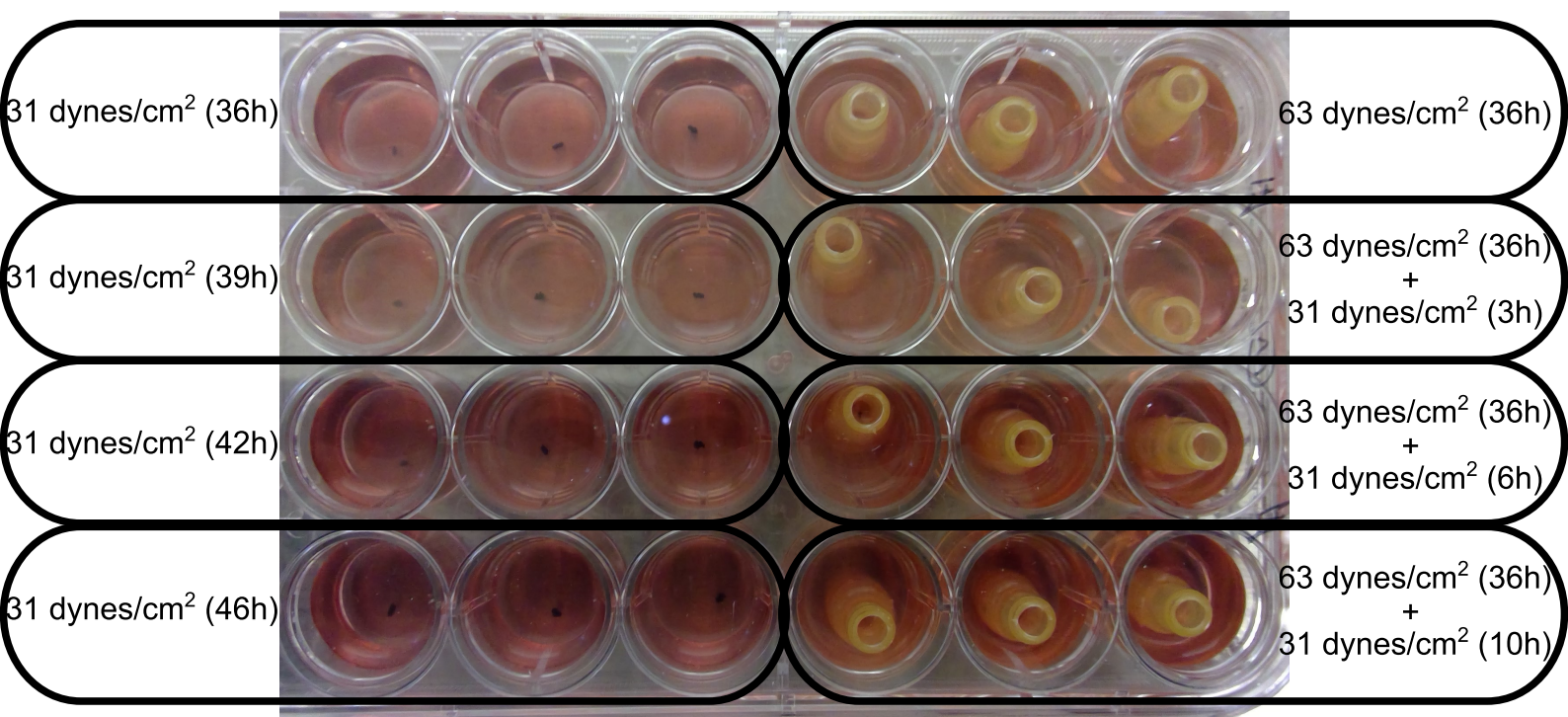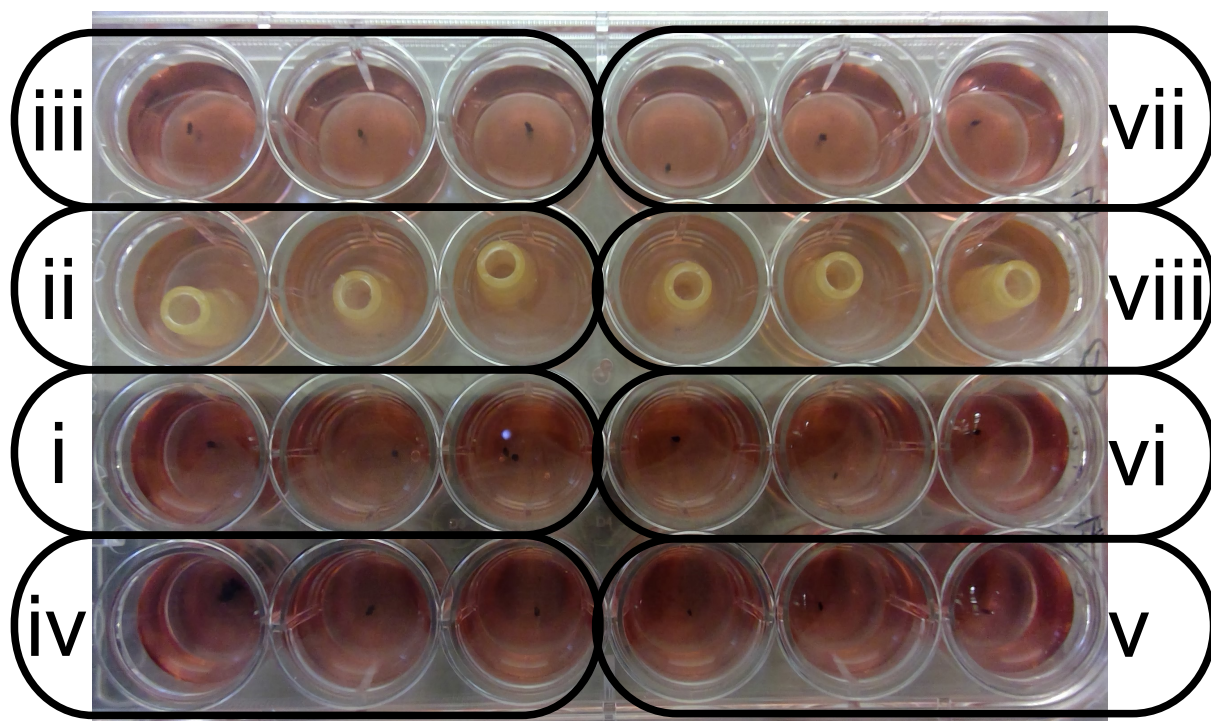

|                                      | i | ii | iii | iv | v | vi | vii | viii |
|--------------------------------------|---|----|-----|----|---|----|-----|------|
| 3μM 18α-GA (2h)                      | - | -  | -   | -  | + | -  | +   | +    |
| DMSO                                 | - | -  | -   | +  | + | +  | +   | +    |
| CCF at 63 dynes/cm <sup>2</sup> (1h) | - | +  | +   | -  | - | +  | +   | +    |
| Loading history                      | - | +  | -   | -  | - | -  | -   | +    |

Supplement: Supplemental Information 1 — Experiment design for RNA extraction and cell morphological measurements. [file peerj-08-10244-s001.pdf]

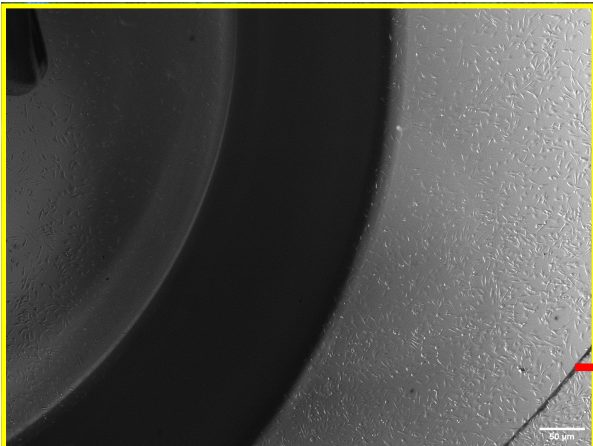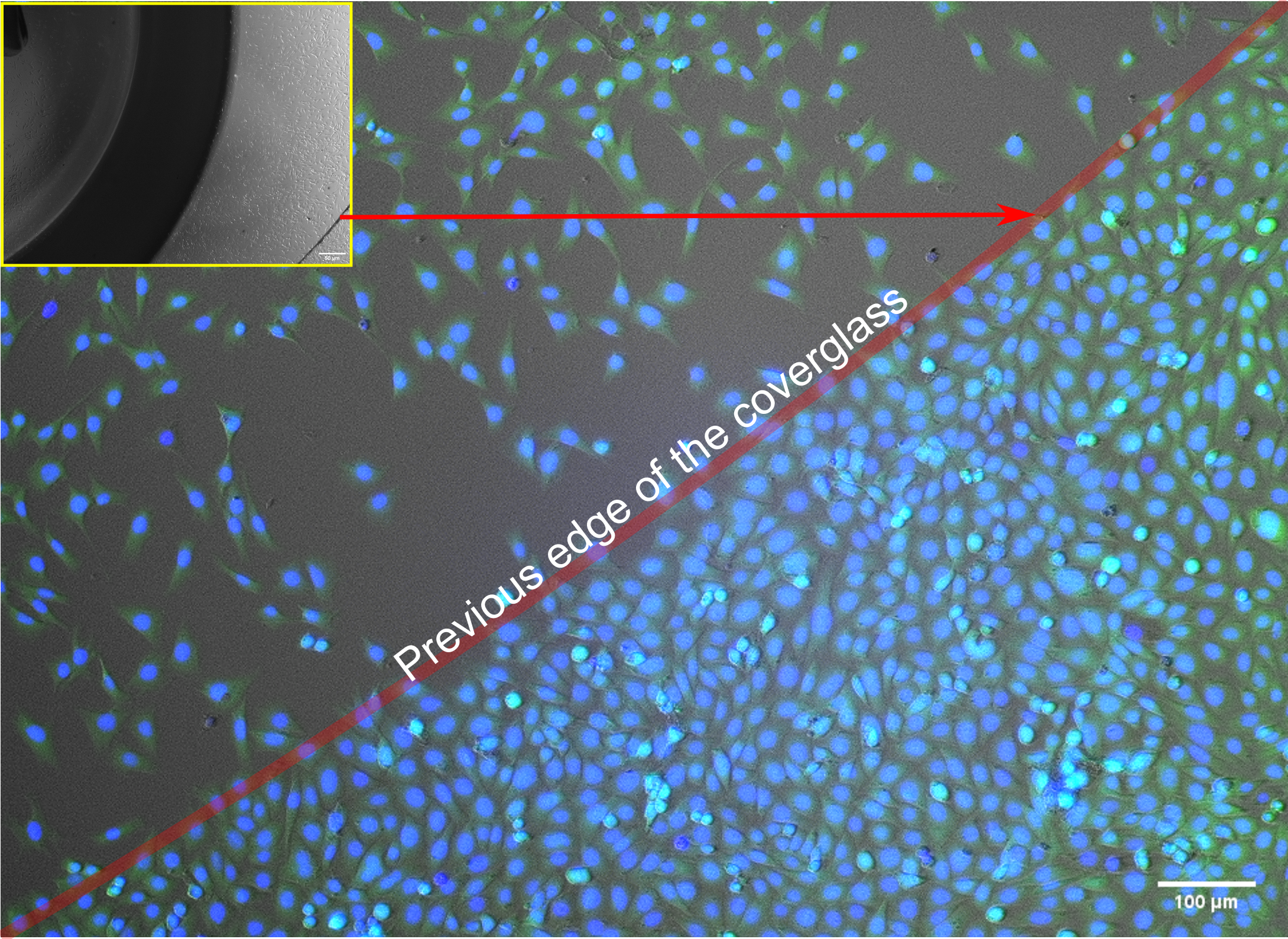

Supplement: Supplemental Information 2 — After 46 h of exposure to 31 dynes/cm2 CCF (MLO-Y4 cells without a loading history). The cover glass was removed when starting the Calcein-AM and Hoechst 33342 staining. [file peerj-08-10244-s002.pdf]
